# Topoisomerase I inhibition in colorectal cancer: biomarkers and therapeutic targets

**DOI:** 10.1038/bjc.2011.498

**Published:** 2011-11-22

**Authors:** D C Gilbert, A J Chalmers, S F El-Khamisy

**Affiliations:** 1 Sussex Cancer Centre, Royal Sussex County Hospital, Eastern Road, Brighton BN2 5BE, UK; 2 Institute of Cancer Sciences, University of Glasgow, Glasgow G12 8QQ, UK; 3 Genome Damage and Stability Centre, University of Sussex, Falmer, Brighton BN1 9RQ, UK; 4 Department of Biochemistry, Faculty of Pharmacy, Ain Shams University, Cairo POB 11566, Egypt

**Keywords:** irinotecan, colorectal cancer, biomarkers, topoisomerase I, TDP1, PARP

## Abstract

The topoisomerase I (Top 1) poison irinotecan is an important component of the modern treatment of colorectal cancer. By stabilising Top 1-DNA complexes, irinotecan generates Top 1-linked DNA single-strand breaks that can evolve into double-strand breaks and ultimately cause cell death. However, cancer cells may overcome cell killing by releasing the stalled topoisomerase from DNA termini, thereby reducing the efficacy of Top 1 poisons in clinics. Thus, understanding the DNA repair mechanisms involved in the repair of Top 1-mediated DNA damage provides a useful tool to identify potential biomarkers that predict response to this class of chemotherapy. Furthermore, targeting these pathways could enhance the therapeutic benefits of Top 1 poisons. In this review, we describe the cellular mechanisms and consequences of targeting Top 1 activity in cells. We summarise preclinical data and discuss the potential clinical utility of small-molecule inhibitors of the key proteins.

## Clinical utility of topoisomerase I (Top 1) poisons

Colorectal cancer remains a significant cause of morbidity and mortality worldwide, with 39 000 new cases per year in the UK and 16 000 deaths. In North America, the figures are 177 000 and 58 000, respectively (http://globocan.iarc.fr/). Despite the development of biological agents targeting EGFR and VEGF signalling, defining subgroups of patients that derive maximal benefit has proved difficult. Combination chemotherapy consisting of 5-fluorouracil paired with either the third-generation platinum compound oxaliplatin or the Top 1 poison irinotecan remains the mainstay of treatment for metastatic disease. The efficacy of irinotecan in metastatic colorectal cancer was demonstrated in clinical trials conducted over a decade ago (Douillard *et al*, 2000; Saltz *et al*, 2000), with response rates to combination regimens of 30–50% and overall survival in some studies approaching 24 months (Fuchs *et al*, 2007). In the treatment-naive population, there is broad equivalence in tumour response between irinotecan and oxaliplatin when combined with 5-fluorouracil (Seymour *et al*, 2007). However, the observation that responses to both irinotecan and oxaliplatin occur in the second line setting after progression on the other drug indicates that individual tumours differ in their sensitivity to these drugs. Biomarkers are therefore required to optimise patient treatment.

Locally advanced rectal cancers are increasingly treated with neoadjuvant chemo-radiotherapy strategies to optimise surgical resection and reduce rates of local and distant relapse. Phase I/II studies incorporating irinotecan, 5FU and radiotherapy in rectal cancer have indicated improved efficacy over 5FU chemo-radiotherapy alone, and have proved to be deliverable in terms of acute toxicity (Glynne-Jones *et al*, 2007; Willeke *et al*, 2007; Gollins *et al*, 2011). Neoadjuvant strategies incorporating oxaliplatin are also being developed, and thus robust predictive markers are required to optimise patient selection and maximise clinical benefit. Beyond its role in colorectal cancer, which will be the main focus of this review, there is also growing interest in the use of irinotecan in small-cell lung cancer, where there is evidence of increased efficacy over etoposide regimens (Lima *et al*, 2010), and a range of other tumour types including glioblastoma.

## Cellular biochemistry of Top 1

The compact and supercoiled nature of the DNA double helix requires topological modification during important cellular processes such as transcription, replication and repair. This modification is conducted by DNA topoisomerases and involves transient cleavage and re-ligation of the double-stranded DNA molecule. Topoisomerases are enzymes that cleave one or both of the sugar-phosphate backbones of double-stranded DNA without altering its chemical composition (hence the term ‘isomerase’). Type I topoisomerases (Top I, Wang, 1971) cut a single strand of DNA to allow relaxation of torsional stresses before re-annealing. Type II topoisomerases (Top II, Gellert *et al*, 1976) incise double-stranded DNA to facilitate the passage of an intact duplex through the gap before rejoining the cut DNA. This mode of catalysis involves an intermediate known as the cleavage complex, which comprises the topoisomerase enzyme attached to the cleaved DNA by a covalent phosphotyrosyl bond. Increased levels of Top I mRNA and protein are seen across human tumours (Husain *et al*, 1994), suggesting increased transcription or mRNA stability, although genomic amplification of Top I in colorectal cancer has been described and correlates with increased RNA and protein expression (Yu *et al*, 2008).

Top I is the target of the camptothecin derivatives irinotecan and topotecan, whereas Top II is targeted by etoposide and anthracyclins. Camptothecin is a naturally occurring cytotoxic quinolone alkaloid (derived from the bark of *Camptotheca acuminata*) that binds to and stalls Top 1 on DNA. Irinotecan is a semisynthetic analogue of camptothecin that is activated by hydrolysis to the active metabolite SN38, which is subsequently metabolised through glucoronidation by uridine diphosphate glucoronosyltransferase 1A1 (UGT1A1) and excreted in the bile. Patients with specific polymorphisms in UGT1A1 (UGT1A1^*^28) have impaired metabolism of SN38 and are predisposed to the major toxicities of irinotecan, which are diarrhoea and myelosuppression, particularly neutropenia (Innocenti *et al*, 2004; O’Dwyer and Catalano, 2006). More recently, it has been suggested that different polymorphisms in UGT1A might also modulate tumour response rates (Cecchin *et al*, 2009).

**Figure 1:**
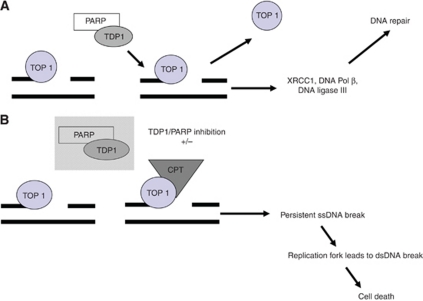
(**A**) Top 1 cleavage complexes are ordinarily removed through TDP1- and PARP-dependent mechanisms, with the ssDNA breaks repaired through XRCC1 and DNA through polymerases/ligases. (**B**) Camptothecin (and irinotecan via SN38) stabilises the cleavage complexes, with the persistent ssDNA breaks converting to dsDNA lesions causing cell death, and expected synergy with TDP1/PARP inhibition.

Irinotecan (predominantly in the form of SN38) binds to the Top I-DNA complex, stabilizing it and preventing re-ligation (Hsiang *et al*, 1985; Hsiang and Liu, 1988). Collision with advancing replication forks results in the formation of double-stranded DNA breaks. These breaks activate cell cycle arrest in G2 phase and, if unrepaired, can cause cell death (Figure 1). The requirement for DNA replication in this cytotoxic mechanism confers a degree of tumour specificity, with the major toxic effects arising in rapidly proliferating normal tissues. However, cell cycle-independent cytotoxicity may also occur through apoptosis, which is thought to be triggered by inhibition of Top I activity during DNA transcription (Morris and Geller, 1996). There is also recent evidence that activation of p38 MAPK may protect cells from irinotecan cytotoxicity (Paillas *et al*, 2011).

Repair of irinotecan-associated DNA damage requires removal of the stalled Top 1 peptide and resolution of the associated DNA break (Figure 1). A number of repair proteins are involved in this process, some of which have clinical potential either as predictive biomarkers or as therapeutic targets. The most important factors will be briefly described in this section of the review.

Excision of the covalently linked topoisomerase is mandatory if subsequent repair steps are to be initiated; this can be achieved either by nonspecific nucleolytic cleavage of DNA, releasing the topoisomerase and a fragment of DNA, or by hydrolytic cleavage of the covalent phosphotyrosyl bond that links the topoisomerase to the DNA termini (reviewed in El-Khamisy, 2011). The prototype enzyme for the latter process was first identified in yeast and named tyrosyl DNA phosphodiesterase 1 (Yang *et al*, 1996). Tyrosyl DNA phosphodiesterase 1 (TDP1) catalyses hydrolysis of the phosphodiester bond between Top 1 and the 3′-phosphate of DNA, allowing resolution of the stalled Top I-DNA complexes (El-Khamisy *et al*, 2005; Interthal *et al*, 2005). In a similar manner, a second protein (TDP2) functions to remove Top II covalently bound to DNA double-strand breaks (Cortes Ledesma *et al*, 2009). Lack of TDP1 is associated with defects in the repair of Top 1-associated DNA strand breaks, and cells deficient in TDP1 accumulate DNA strand breaks when incubated with camptothecin.

*In vitro*, TDP1 can process a variety of 3′-oxidative termini, including 3′-phosphoglycolate moieties that are a common feature of DNA breaks induced by ionising radiation (IR; Zhou *et al*, 2005, 2009; El-Khamisy *et al*, 2007; Chiang *et al*, 2010). This observation points to a requirement for TDP1 for effective resolution of DNA damage associated with both Top 1 inhibition and IR, and indicates a potentially critical role for TDP1 in the cellular response to irinotecan-based chemoradiation. The clinical implications of this will be discussed later.

Poly(ADP-ribose) polymerase (PARP) also influences repair of Top I-mediated DNA damage. Inhibition of PARP sensitises cells to camptothecin, primarily by delaying DNA repair (Smith *et al*, 2005). As PARP inhibition does not confer additional sensitivity to camptothecin in TDP1 knockout cells (Zhang *et al*, 2011), it has been suggested that PARP and TDP1 are components of a single repair pathway for Top I–DNA complexes. In the same study, XPF and ERCC1 were also shown to be involved in repair of camptothecin-induced DNA damage; however, unlike *TDP1*, knockdown of *XPF* was synergistic with PARP inhibition in terms of camptothecin cytotoxicity (Zhang *et al*, 2011). PARP/TDP1 and XPF-ERCC1 may therefore comprise alternative pathways for repairing Top 1-mediated DNA damage.

Another candidate that has a role during the repair of Top 1 breaks is Aprataxin (APTX)*. APTX* is the gene mutated in ataxia-ocular apraxia 1 (Moreira *et al*, 2001) and encodes a protein involved in the repair of single- (SSB) and double-stranded (DSB) DNA breaks. There is evidence that APTX participates in the repair of CPT-induced DNA damage (Mosesso *et al*, 2005), and a synergy between the actions of TDP1 and APTX has also been reported during the repair of specific types of DNA breaks (El-Khamisy *et al*, 2009).

Approximately 5% of colorectal cancers are associated with hereditary non-polyposis colon cancer, an inherited cancer predisposition syndrome caused by germ-line alteration of mismatch repair genes (MMR). Moreover, around 15% of sporadic cases demonstrate somatic loss of function of one or more MMR genes (most commonly *MSH2* or *MLH1*). The MMR proteins recognise errors in the base sequence of DNA that occur during DNA replication (insertions, deletions or substitutions) and facilitate excision of the mismatched strand and restoration of base fidelity. Loss of function of one or more of these proteins results in microsatellite instability (MSI) – abnormally long or short microsatellites (repeated sequences of DNA) – which serves as a genetic signature for this phenotype. MMR-defective colorectal cancer lines exhibit increased sensitivity to CPT *in vitro*, which is reversed when wild-type gene expression is restored (Jacob *et al*, 2001). MMR proteins may also have additional roles in DSB repair and induction of apoptosis in response to DNA damage, and it is hypothesised that these actions may contribute to modulating the response to Top 1 response to topoisomerase poisons detailed below.

## Therapeutic targets in the context of Top 1 inhibition

Much of our understanding of the proteins involved in repairing Top I cleavage complexes is derived from experimental strategies in which inhibition of those proteins potentiates the DNA damage sustained. Such proteins therefore constitute targets for therapeutic interventions aimed at improving the clinical efficacy of irinotecan.

Cell line and xenograft data demonstrate the potentiating effect of PARP inhibition on the cytotoxicity of irinotecan (Miknyoczki *et al*, 2003; Calabrese *et al*, 2004; Smith *et al*, 2005). Phase I studies of a number of PARP inhibitors, for example, veliparib (Abbott Laboratories, Abbott Park, IL, USA), iniparib (Sanofi-Aventis, Surrey, UK) and olaparib (AstraZeneca, London, UK), in combination with irinotecan are underway. The radiosensitising effects of PARP inhibition are also well documented (Calabrese *et al*, 2004; Chalmers *et al*, 2010; Efimova *et al*, 2010), and clinical trials in various tumour sites are in development (Verheij *et al*, 2010). Locally advanced rectal cancer may therefore provide an ideal opportunity to test combinations of irinotecan, radiotherapy and PARP inhibitors. Of critical importance will be whether meaningful improvements in tumour response can be achieved without unacceptable exacerbation of normal tissue toxicities, particularly bone marrow suppression and diarrhoea.

The rationale for developing inhibitors of TDP1 for subsequent combination with Top 1 poisons is similarly robust. *In vitro*, cells deficient in TDP1 accumulate an excess of DNA strand breaks when incubated with camptothecin (El-Khamisy *et al*, 2005; Interthal *et al*, 2005) or exposed to IR (El-Khamisy *et al*, 2007). This dual activity makes inhibition of TDP1 a compelling target for clinical studies in combination with Top I inhibitors and radiotherapy, especially when viewed in the context of the early success of PARP inhibition in clinical practice. Beyond its use in the management of colorectal cancer, the recently demonstrated activity of irinotecan in small-cell lung cancer and glioblastoma may give TDP1 a broader utility. Combining TDP1 inhibitors with topotecan in ovarian cancer may also prove synergistic. However, given similarities in function and pathways at a cellular level, it remains to be seen how similar or different PARP and TDP1 inhibition will prove and whether inhibiting both together would prove synergistic or mutually redundant.

## Potential as predictive biomarkers

As described previously, there are a number of therapeutic options available for patients with colorectal cancer, and patient selection is a critical process that is currently sub-optimal. Our increasing knowledge of the mechanisms determining sensitivity to Top 1 inhibitors raises the possibility that some of the key molecules described above will have utility as biomarkers that predict response of tumours to treatment.

### Top 1

As the cytotoxic effects of topoisomerase poisons are dependent on stabilisation of the topoisomerase–DNA complex, it is reasonable to predict that cellular sensitivity to these agents will be modulated by absolute Top I levels, although cell lines containing Top I mutations that alter Top I DNA or camptothecin interaction have been described that confer resistance to camptothecin (Li *et al*, 1996; Gongora *et al*, 2011). Repeated exposure of colorectal cancer xenografts to camptothecin resulted in downregulation of Top I levels (Giovanella *et al*, 1989) and the same effect has been observed in peripheral blood mononuclear cells after treatment with topotecan (Hochster *et al*, 1997). Clinically, tumour expression of Top I decreased (between pretreatment biopsy and subsequent surgical specimen) following neoadjuvant treatment of rectal cancer with chemoradiation comprising irinotecan and 5FU (Horisberger *et al*, 2009).

Top I is highly expressed in around half of the colorectal cancers, with one study demonstrating higher levels in rectal cancers (Boonsong *et al*, 2002). The observed broad range of expression supports the hypothesis that Top I expression will predict response to irinotecan. It has been suggested that higher levels of Top I expression may predict response to irinotecan-containing neoadjuvant chemoradiation in rectal cancer (Horisberger *et al*, 2009). In addition, the results of the MRC FOCUS study of 1313 patients with metastatic colorectal cancer indicated that tumours with moderate or high levels of Top I expression as determined by immunohistochemistry showed the greatest benefit from adding irinotecan or oxaliplatin to 5FU in the first-line metastatic setting (Braun *et al*, 2008). However, subsequent data from the similar ‘CAIRO’ study from the Dutch Colorectal Cancer Group (Koopman *et al*, 2007) failed to replicate these findings, with no association seen between Top 1 expression (by immunohistochemistry) and response to irinotecan and capecitabine in 545 patients (Koopman *et al*, 2009). These apparently contradictory findings suggest that although absolute Top I expression levels may play a part, it is likely that additional molecules contribute to irinotecan sensitivity in the clinic.

### TDP1

The critical role of TDP1 in determining cellular responses to irinotecan makes it a promising biomarker. A number of studies have investigated polymorphisms in genes involved in irinotecan/Top 1-related DNA repair and response to treatment, and some of these have included *TDP1*. In one such study, 107 patients treated with irinotecan were screened for host polymorphisms in *PARP*, *TDP1*, *Top 1* and *XRCC1* (Hoskins *et al*, 2008). Univariate analysis suggested that specific polymorphisms in *TDP1* and *XRCC1* were linked with response to irinotecan, but on multivariate analysis only *XRCC1* remained significant.

The available data indicate that *TDP1* expression is increased in colorectal tumour samples compared with paired normal tissue (Yu *et al*, 2005). In the most relevant study to date, 52 metastatic colorectal cancer specimens were analysed by RT–PCR for expression of 24 genes hypothesised to be associated with response to irinotecan. *TDP1* was one of eight genes (including *ERCC1* – see above) that showed significantly higher levels of expression in tumours than in normal tissue. Expression of *TDP1* grouped with other genes involved in DNA repair. Interrogation of oncomine (www.oncomine.org) supports this finding, with *TDP1* expression levels appearing to be broadly increased in colorectal cancer specimens. Several microarray expression profiles for rectal adenocarcinomas have been published (NCBI GEO, EBI), but only one of these used a platform that included a probe for *TDP1* (Snipstad *et al*, 2010). Our analysis of these data demonstrates increased levels of *TDP1* in rectal cancers compared with normal tissues.

As detailed above, it has also been shown that TDP1 has a role in the repair of SSBs induced by IR (El-Khamisy *et al*, 2007). Specifically, cells deficient in TDP1 exhibit delayed repair of SSBs induced by IR (Katyal *et al*, 2007; Chiang *et al*, 2010). Although the cytotoxic effects of IR are predominately mediated through double-strand breaks, unrepaired SSBs can be converted to DSBs during DNA replication. This raises the intriguing possibility that TDP1 could be a dual biomarker for sensitivity to both irinotecan and radiotherapy. Although there is no published data to substantiate this claim, high quality tissue is available from several clinical trials that have tested irinotecan-based chemoradiation regimes, and these samples are currently being analysed for expression of TDP1 and other relevant DNA repair genes and proteins.

Finally, *in vitro* experiments using the PARP inhibitor ABT-888 (Zhang *et al*, 2011) show no further enhancement of camptothecin cytotoxicity in cell lines lacking TDP1, suggesting that PARP and TDP1 comprise a common repair pathway. Although this supports the rationale for either (but not both together) as therapeutic targets in potentiating topoisomerase poisons, it is possible that increased TDP1 expression levels might prove a biomarker in predicting benefit from the addition of PARP inhibitors to irinotecan or radiotherapy.

### APTX

*In vitro* studies of colon cancer cell lines have shown an association between APTX expression levels and sensitivity to camptothecin (Mariadason *et al*, 2003), and there is also evidence to suggest that APTX modulates response to irinotecan in metastatic colorectal cancer, with higher protein expression associated with a lower likelihood of response. Tumour blocks from 135 patients with metastatic disease treated with a variety of irinotecan/5FU combination regimens were probed for APTX using immunohistochemistry (Dopeso *et al*, 2010). With a median follow-up of 4.6 years, patients with low levels of APTX had improved progression-free and overall survival (PFS 9.2 *vs* 5.5 months *P*=0.03, OS 36.7 *vs* 19 months *P*=0.008). These promising data require validation, but demonstrates the potential value of this class of biomarker.

### MMR

Mismatch repair-deficient colorectal cancers have been reported to be resistant to 5FU (Ribic *et al*, 2003), but more recent evidence indicates that they may be sensitive to irinotecan. In the adjuvant setting, CALGB 89803 randomised 1264 patients with stage III colon cancer to weekly 5FU/leucovorin±irinotecan. Of all, 723 cases were retrospectively genotyped for MSI, and MMR protein expression was analysed by immunohistochemistry (Bertagnolli *et al*, 2009). Tumours with evidence of MMR deficiency showed improved 5-year disease-free survival when treated with irinotecan (0.76 *vs* 0.59, *P*=0.03), a difference that was not observed in the 5FU-treated arm. This effect has also been documented in the metastatic setting (Fallik *et al*, 2003). Here, 72 patients treated with irinotecan-containing regimens were analysed for loss of expression of hMLH1 and hMSH2 and genotyped for microsatellite instability. Four out of seven tumours with high levels of MSI responded to irinotecan as opposed to seven out of sixty-five patients with low-level MSI (*P*=0.009). However, unlike Top I, MLH1/MSH2 immunohistochemical analysis was not able to predict response to irinotecan (or oxaliplatin) within the FOCUS study (Braun *et al*, 2008), although with only 4.4% samples showing evidence of impaired mismatch repair the statistical power was low. As molecular subtyping of colorectal cancer improves, it is likely that MMR-deficient tumours will acquire specific treatment protocols. Current understanding of DNA repair mechanisms would place irinotecan at the centre of these, but more clinical data are required before such protocols are adopted.

### Biomarkers of toxicity

Given the equivalent first-line efficacy of oxaliplatin and irinotecan regimens, the ability to predict toxicity would be of value in individualising treatment decisions. Here, germ-line polymorphisms in the genes discussed in this review may be more relevant than variations in tumour expression. The previously described study (Hoskins *et al*, 2008) genotyped 107 metastatic CRC patients treated with irinotecan regimens for single-nucleotide polymorphisms (SNP) in *Top 1*, *CDC45L*, *NFKB1*, *PARP1*, *TDP1* and *XRCC1*. These SNPs were tested for association with the most frequent and significant side effects of irinotecan, namely grade three out of four diarrhoea and neutropenia. In univariate analysis, SNPs in both *Top 1* and *TDP1* were associated with grade three out of four neutropenia. However, multivariate analysis failed to demonstrate significant association, and the same authors failed to replicate these findings in a separate sample set (Hoskins *et al*, 2009). However, neither study was powered to detect relatively small effects, and consideration of the overlapping pathways involved in determining irinotecan response suggests that any modulation of toxicity is likely to be multifactorial.

## Clinical application of potential biomarkers

There is increasing awareness of the potential value of molecular pathology in clinical decision making, and colorectal cancer is at the forefront of this vogue. The MRC FOCUS 3 trial is currently testing the feasibility of such a strategy in a study that stratifies patients with metastatic colorectal cancer into treatment groups based on Top 1 I levels in their tumour specimens (http://www.ctu.mrc.ac.uk/). Drawing on molecular data from the FOCUS study described above (Braun *et al*, 2008), and using combination irinotecan and 5FU as a control regimen, patients with low Top I-expressing tumours will be randomised to omit the irinotecan (i.e., receive 5FU alone), whereas tumours with high Top I will be randomised to add oxaliplatin to irinotecan/5FU. A further randomisation will be determined by the mutation status of *KRAS* and *BRAF*, with the addition of cetuximab being tested if *KRAS/BRAF* are both wild type and bevacizumab if either are mutated. If successful, this ambitious study will be extremely informative both from a clinical perspective and as an indicator of the feasibility of individualising treatment by virtue of molecular testing.

Increasing application of irinotecan in the neoadjuvant treatment of rectal cancer (Gollins *et al*, 2011) may provide opportunities for testing a range of the potential biomarkers discussed in this review. MRI and pathological response at definitive surgery provide robust and quantitative early-outcome measures, and the availability of pre- and post-treatment tissue samples makes this an ideal setting in which to investigate new drug combinations and associated biomarkers.

## Potential utility beyond topoisomerase inhibitors

There is increasing interest in the use of small-molecule inhibitors of DNA repair enzymes to overcome resistance to conventional cytotoxic agents that kill cells predominantly by damaging DNA. PARP inhibitors are at the forefront of this field, and several clinical trials combining PARP inhibitors with radiotherapy and/or cytotoxic drugs are either underway or in development. As previously highlighted, there are functional parallels between TDP1 and PARP, with TDP1 having a role in the resolution of SSBs induced by Top 1 poisons and by ionising radiation. Hence, there is a biological rationale for combining TDP1 inhibitors with radiotherapy (El-Khamisy *et al*, 2007), either alone or in the context of chemoradiation schedules. Although relatively little cancer-specific research has been conducted, TDP1 is known to be expressed in a variety of tumour types (Liu *et al*, 2007). In addition to the compelling evidence for TDP1 as a therapeutic target in the treatment of rectal cancer, it is reasonable to predict that ongoing research will identify whether additional therapeutic applications exist for combination treatments comprising TDP1 inhibitors.

## Conclusions

Although decades of basic scientific research has yielded a number of anti-cancer drugs that target signal transduction pathways, only recently has there been a resurgence of interest in understanding and exploiting the cellular mechanisms of DNA repair. This new knowledge promises to better explain clinical responses to conventional cytotoxic agents including radiotherapy, and to reveal biomarkers predictive of response and resistance (Table 1). Specifically, the mechanisms for repairing topoisomerase-associated DNA breaks that accumulate following treatment with Top I poisons, such as irinotecan, comprise proteins that can be targeted to modulate sensitivity to these agents. TDP1 has well-characterised roles in the repair of DNA-Top I intermediates and radiation-induced DNA breaks and shows significant promise as a biomarker. Furthermore, the clinical development of PARP inhibitors has demonstrated that this understanding can identify therapeutic targets, inhibitors of which might realistically be combined with irinotecan to yield clinically significant improvements in tumour response rates.

The promise of the biomarkers described in this review should be comprehensively assessed by translational work on the plethora of clinical studies that have used irinotecan in colorectal cancer (and beyond). Retrospective work, however, will require the cooperation of treating departments in collecting meaningful sample sets. The development of trials that begin to match treatment arms to underlying molecular characteristics (e.g., FOCUS3) should be widely supported and further developed. Combining an improved molecular understanding of individual tumours with specific adjunctive therapies, Top 1 will remain a key target in the treatment of colorectal cancer.

## Figures and Tables

**Table 1:** Candidate therapeutic targets and biomarkers of response to irinotecan derived from an improved understanding of topoisomerase I activity and subsequent DNA repair mechanisms

	**Potential clinical utility**		
**Molecule**	**Therapeutic target**	**Biomarker of response**	**Reference**
Top I	Mechanism of action of Irinotecan	Yes	Braun *et al*, 2008
TDP1	Yes	Proposed	El-Khamisy *et al*, 2005, 2007
PARP	Yes	?	Smith *et al*, 2005; Zhang *et al*, 2011
Aprataxin	?	Yes	Dopeso *et al*, 2010
Mismatch repair	?	Yes	Bertagnolli *et al*, 2009

Abbreviations: PARP=poly(ADP-ribose) polymerase; TDP1=tyrosyl DNA phosphodiesterase 1; Top I=type I topoisomerases.
